# Effects of Urotensin II Receptor Antagonist, GSK1440115, in Asthma

**DOI:** 10.3389/fphar.2013.00054

**Published:** 2013-04-29

**Authors:** Alison Portnoy, Sanjay Kumar, David J. Behm, Kelly M. Mahar, Robert B. Noble, John P. Throup, Steven F. Russ

**Affiliations:** ^1^Virtual Proof of Concept Discovery Performance UnitGlaxoSmithKline, King of Prussia, PA, USA; ^2^Heart Failure Discovery Performance UnitGlaxoSmithKline, King of Prussia, PA, USA; ^3^Clinical Pharmacology Modeling and SimulationGlaxoSmithKline, King of Prussia, PA, USA; ^4^Quantitative SciencesGlaxoSmithKline, Collegeville, PA, USA; ^5^Virtual Proof of Concept Discovery Performance UnitGlaxoSmithKline, Research Triangle Park, NC, USA

**Keywords:** urotensin II, asthma, receptor antagonist

## Abstract

**Background:** Urotensin II (U-II) is highly expressed in the human lung and has been implicated in regulating respiratory physiology in preclinical studies. Our objective was to test antagonism of the urotensin (UT) receptor by GSK1440115, a novel, competitive, and selective inhibitor of the UT receptor, as a therapeutic strategy for the treatment of asthma.

**Methods:** Safety, tolerability, and pharmacokinetics (PK) of single doses of GSK1440115 (1–750 mg) were assessed in a Phase I, placebo controlled study in 70 healthy subjects. In a Phase Ib study, 12 asthmatic patients were randomized into a two-period, single-blind crossover study and treated with single doses of 750 mg GSK1440115 or placebo and given a methacholine challenge.

**Results:** Administration of GSK1440115 was safe and well-tolerated in healthy subjects and asthmatic patients. In both studies, there was a high degree of variability in the observed PK following oral dosing with GSK1440115 at all doses. There was a marked food effect in healthy subjects at the 50 mg dose. In the presence of food at the 750 mg dose, the time to maximal concentration was between 2 and 6 h and the terminal half-life was short at approximately 2 h. All asthmatic patients maintained greater than the predicted concentration levels necessary to achieve predicted 96% receptor occupancy for ≥3 h (between 4 and 7 h post-dose). There were no apparent trends or relationships between the systemic plasma exposure of GSK1440115 and pharmacodynamic endpoints, PC_20_ after methacholine challenge and FEV1, in asthmatics.

**Conclusion:** While GSK1440115 was safe and well-tolerated, it did not induce bronchodilation in asthmatics, or protect against methacholine-induced bronchospasm, suggesting that acute UT antagonism is not likely to provide benefit as an acute bronchodilator in this patient population.

## Introduction

Asthma is characterized by blockage, narrowing, or constriction of airways resulting in breathing difficulties with symptoms such as coughing, wheezing, shortness of breath, or rapid breathing, and chest tightness. It is a chronic disease affecting over 20 million Americans and is either allergic (non-intrinsic triggered by inhaled allergens) or non-allergic (triggered by intrinsic factors). Despite available therapies, there is still an unmet medical need for a safe, orally administered bronchodilator, particularly since long-acting β agonists are undergoing increased scrutiny for potential deleterious effects in this population (Sears, 2011).

Human urotensin II (hU-II) is a cyclic undecapeptide whose actions are mediated by a specific cell surface G-protein-coupled receptor, the urotensin II (U-II) receptor, UT (Ames et al., 1999; Alexander et al., 2009). U-II was originally described as a potent vasoconstrictor (Douglas et al., 2000). Since then, there has been increasing evidence for U-II’s role in regulating respiratory physiology. It is highly expressed in the lung (Zhang et al., 2002; Qi et al., 2004), which is a major source of U-II in humans (Russell et al., 2003). Similarly, U-II receptor expression has been localized to rat airway smooth muscle cells where it functions as a growth factor (Chen et al., 2004; Zhang et al., 2012). Furthermore, U-II can mediate smooth muscle contraction in isolated respiratory tract and pulmonary arteries in primates (Douglas et al., 2000; Hay et al., 2000; MacLean et al., 2000). Most recently, U-II has been implicated in the remodeling of airways in asthmatic rats (Liang et al., 2010). Finally, potent U-II receptor antagonists can block U-II-induced contraction of isolated cat trachea muscle (Behm et al., 2005).

GSK1440115 is a potent (K_i_ of 2.3 and 4.6 nM for recombinant and native human UT receptors, respectively), competitive and selective inhibitor of UT which mediates hU-II-induced systemic pressor response in anesthetized cats (Behm et al., 2010). Given the evidence for a role for U-II in the lung, we tested whether UT antagonism, as mediated through GSK1440115, would be an effective therapeutic strategy for the treatment of asthma. In this report, we present the results of the first two clinical studies conducted with GSK1440115. The first trial was a Phase I first-time-in-human (FTiH) study to evaluate the safety, tolerability, and pharmacokinetics (PK) of rising single doses of GSK1440115 in healthy volunteers. The results of the FTiH study were used to design the subsequent Phase Ib proof-of-mechanism study that evaluated the activity of GSK1440115 in asthmatic subjects.

## Materials and Methods

### Study design and participants

#### Phase I trial – first-time-in-human (healthy volunteers)

The FTiH trial was a randomized, single-blind (subjects and investigator), placebo controlled, dose escalation study in healthy volunteers (ClinTrials.gov identifier: NCT01202214). The sponsor was unblinded to study treatment. At least eight subjects were enrolled in each of the nine cohorts, such that at least six subjects randomly received the same dose of GSK1440115 and at least two subjects received placebo. The study was composed of three periods for all subjects (screening, treatment, and follow-up). Safety, PK and pharmacodynamics were assessed throughout the study.

Eligible subjects included healthy adult males and females (of non-child-bearing potential) 18–65 years of age, inclusive. Subjects had AST, ALT, alkaline phosphatase, and bilirubin ≤1.5 × ULN (isolated bilirubin >1.5 × ULN was acceptable if bilirubin was fractionated and direct bilirubin was <35%) and QTcB or QTcF <450 ms; or QTc <480 ms in subjects with Bundle Branch Block (ClinTrials.gov identifier: NCT01424280).

The initial oral dose of GSK1440115 in the FTiH study was calculated using multiple independent approaches including the United States Food and Drug Administration (FDA) Guidance, prediction of intrinsic human clearance using *in vitro* data from human microsomes (with and without unbound fraction) (FDA Guidance, 2005), and allometric and multiple species scaling based on intravenous PK data from preclinical species. The human starting doses were calculated using the lowest no adverse event level (NOAEL) dose/exposure in the 28-days repeat dose toxicology studies, and then applied a safety factor of 10-fold, or a more conservative safety factor of 100-fold. The lowest NOAEL exposure was the 1000 mg/kg dose in male rats with an area under the concentration curve (AUC) of 25.6 μg h/ml and a maximum observed concentration (*C*_max_) of 10.1 μg/ml. The NOAEL exposure in female rats and male/female dogs were higher, so the male rat NOAEL exposure was considered the most conservative upper limit of the safety window, and was the limiting exposure for this study. All starting dose considerations indicated that a 1 mg dose would be a safe starting dose for this study in healthy human volunteers with a wide anticipated safety margin to the NOAEL in 28 days toxicology studies. Single oral doses of GSK1440115 from 1 to 750 mg were evaluated in humans.

Preliminary safety and PK data from each cohort were reviewed prior to dose escalation and were used to determine the dose administered in the subsequent cohort. The first five cohorts of subjects were dosed in a fasted state according to the original protocol. Evaluation of the PK data from these early cohorts did not show dose proportional increases in systemic exposure which was thought to be due to the limits of drug dissolution or solubility. Since *in vitro* data with simulated gastric fluids suggested that solubility in a fed environment would be enhanced, the protocol was amended such that all subsequent dosing cohorts received the study medication in the presence of food (high fat meal).

#### Phase Ib trial – proof-of-mechanism in asthmatic subjects

**Figure 1:**
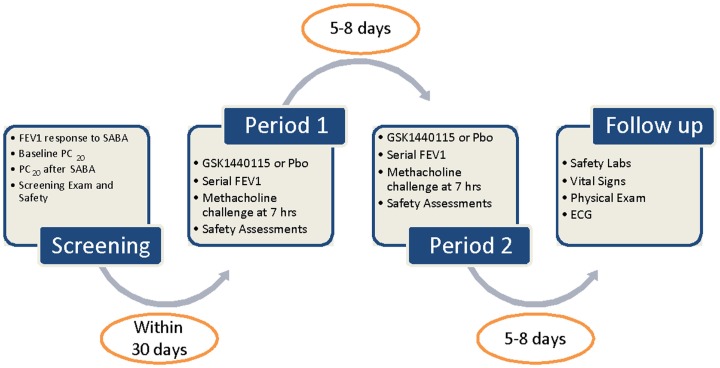
**Study schematic**. SABA, short acting β2 agonist.

The second trial conducted was a two-period, single-blind (subjects and investigator only), randomized crossover study in a single cohort of asthmatics given single 750 mg oral doses of GSK1440115 and placebo as shown in Figure 1 (ClinTrials.gov identifier: NCT01424280). The sponsor was unblinded to study treatment. The 750 mg dose of GSK1440115 was chosen based on its observed safety and PK profiles in the FTiH study and PK/PD modeling which predicted >96% receptor occupancy (RO) would likely be maintained for several hours following dosing. Subjects were randomized to participate in two treatment periods in which a single oral dose of either GSK1440115 or placebo was administered. Each treatment period required two overnight stays at a clinical unit. The treatment periods occurred 5–8 days apart.

Eligible subjects included males and females (non-child-bearing potential) between 18 and 65 years of age with a documented history of bronchial asthma within the British Thoracic Society guideline step 1–3, diagnosed at least 3 months prior to the screening visit, but otherwise healthy. Eligibility also included the following: best forced expiratory volume in 1 s (FEV1) of >70% predicted normal value; hypersensitivity to methacholine such that a baseline provocative concentration of methacholine causing a 20% fall in FEV1 (PC_20_) was ≤4 mg/ml; increase in PC_20_ of at least two dilutions compared to baseline in the presence of inhaled β-agonist in response to a methacholine challenge; average QTcF <450 ms; or QTc <480 ms in subjects with Bundle Branch Block; AST and ALT <2 × ULN; alkaline phosphatase and bilirubin ≤1.5 × ULN.

Each of the two individual studies were conducted at separate single sites in Australia according to the ethical principles of “good clinical practice” (GCP) and the Declaration of Helsinki after obtaining a written informed consent from each subject. The protocol and amendments were all approved by the local institutional review board (IRB).

### Pharmacokinetic assessments

In the Phase I, FTiH study, blood samples for PK analysis were collected at predose (0), 0.5, 1, 2, 4, 8, 12, 18, and 24 h post-dose and a single trough sample on day 3 (approximately 8:00 AM). In the Phase Ib proof-of-mechanism study, blood samples for PK analysis were collected at predose (0), 2, 4, 6, 7, 12, and 24 h post-dose. Concentrations of GSK1440115 were determined in plasma samples using a validated analytical method based on protein precipitation followed by UHPLC/MS/MS analysis. The lower limit of quantification (LLQ) for GSK1440115 was 1 ng/ml using a 50 μL aliquot of acidified human plasma with a higher limit of quantification (HLQ) of 1000 ng/ml.

### Pharmacodynamic assessments

In the Phase Ib study with asthmatic subjects, bronchodilation effect via serial spirometry (FEV1) measurements and via attenuation of bronchoconstriction during a methacholine challenge test were measured. Serial FEV1 measurements were performed at predose and hourly up to 7 h following dosing. A methacholine challenge test occurred at 7 h post-dose. The β-agonist, ventolin, was administered (via spacer) and additional FEV1 assessments were performed to document washout and responsiveness after the methacholine challenge test.

### Statistical analyses

The primary objectives of the FTiH study were to describe the safety, tolerability, and PK in healthy volunteers following single and repeat oral doses of GSK1440115. The primary objective of the Phase Ib, proof-of-mechanism study was to assess the effectiveness of a single oral dose of GSK1440115 to protect against the bronchoconstrictive response induced during a methacholine challenge test in asthmatic subjects who were methacholine responders. The secondary objective for the proof-of-mechanism study was to assess the bronchodilation of GSK1440115 compared to placebo as determined by changes in FEV1; to describe the safety and tolerability; and to characterize the PK of GSK1440115.

For all safety data, summaries of actual value and changes from baseline in the following parameters were generated: vital signs [systolic blood pressure (SBP), diastolic blood pressure (DBP), mean arterial blood pressure (MAP), and pulse rate], ECG values (ventricular rate, intervals of PR, QRS, QT, and QTc), clinical chemistry and hematology values.

For PK data, listings of all derived parameters were generated. For *C*_max_, *t*_max_, AUC_0–τ_, AUC_0–∞_, t^1/2^, R, and λz, the following summary statistics were calculated for each dose: n, arithmetic mean, standard deviation, coefficient of variation, median, minimum, maximum, geometric mean, standard deviation of logarithmically transformed data, and 95% confidence interval for the geometric mean. For Cτ, regular descriptive statistics and 95% confidence intervals about the arithmetic mean was calculated for each dose. The PK-dose relationship was examined graphically by plotting *C*_max_, AUC_0–∞_, and AUC_0–τ_ as a function of the dose levels administered. In practice, at least three dose levels were required to assess dose proportionality.

For pharmacodynamic data, the methacholine PC_20_ for each treatment was summarized categorically showing the counts and percents for each dilution level. FEV1 was analyzed as a continuous variable by treatment for each time point from 0 to 7 h (up to the time of the methacholine challenge). An exploratory analysis was performed to assess the relationship between GSK1440115 exposures and the primary and secondary pharmacodynamic parameters.

## Results

### Demographics

In the FTiH study, 70 subjects were randomized to receive a single dose of study medication (1–750 mg GSK1440115 or placebo) across nine cohorts. All subjects were males and generally well-matched demographically across all dose groups (Table 1).

**Table 1:** **Summary of demographics in phase I and Ib trials – total population**.

Parameter	Phase I (FTiH) study in healthy subjects (*N* = 70)	Phase Ib study in asthmatic subjects (*N* = 12)
Age (year)
Mean	26.7	28.3
Min, Max	19, 63	19, 38
Sex – n (%)
Male	70 (100.0)	10 (83.3)
Female	0 (0.0)	2 (16.7)
Race – n (%)
White	64 (91.4)	11 (91.7)
Asian	6 (8.6)	1 (8.3)
Other	0 (0.0)	0
Height (cm)
Mean	179.1	178.7
Min, Max	166, 198	167, 192

A total of 12 subjects with asthma were randomized and treated in the Phase Ib study with similar demographics in both treatment groups (750 mg GSK1440115 or placebo; Table 1).

### Safety

In the FTiH study, the frequency of reported AEs was low and similar to placebo across all doses of GSK1440115 administered (1–750 mg) in the fed or fasted states (Table 2). All reported AEs were experienced by single subjects, except the AE of upper respiratory tract infection that was reported by two subjects in the placebo group. The most frequently reported AEs across all regimens, including placebo, were upper respiratory tract infection, headache, and orthostatic hypotension. There were no deaths or serious AEs during this study. There were no clinically significant findings in clinical laboratory, or ECGs in this study. There were no dose-related trends in changes in SBP, DBP, MAP, and heart rate (HR), or peak expiratory flow.

**Table 2:** **Summary of all adverse events (related and unrelated) in ≥2 subjects – phase I study in healthy volunteers**.

Adverse event	Number of subjects reporting adverse events										
	Fast/Fed	GSK1440115 fasted					GSK1440115 fed				
	Placebo	1 mg	5 mg	15 mg	50 mg	100 mg	50 mg	150 mg	450 mg	750 mg	Total
	*N* = 18	*N* = 6	*N* = 6	*N* = 6	*N* = 4	*N* = 6	*N* = 6	*N* = 6	*N* = 6	*N* = 6	*N* = 70
Subjects with any AE	6 (33%)	2 (33%)	3 (50%)	2 (33%)	1 (25%)	3 (50%)	1 (17%)	2 (33%)	2 (33%)	2 (33%)	24 (34%)
Upper respiratory tract infection	2 (11%)	1 (17%)	0	1 (17%)	0	1 (17%)	0	0	0	0	5 (7%)
Headache	**1 (6%)**	0	0	**1(17%)**	0	**1 (17%)**	0	**1 (17%)**	0	0	**4 (6%)**
Orthostatic hypotension	**1 (6%)**	0	0	0	**1 (25%)**	0	0	0	0	**1 (17%)**	**3 (4%)**
Dizziness	**1 (6%)**	0	0	0	0	0	0	0	**1 (17%)**	0	**2 (3%)**
Tremor	1 (6%)	0	1 (17%)	0	0	0	0	0	0	0	2 (3%)
Presyncope	0	0	1 (17%)	0	0	0	1 (17%)	0	0	0	2 (3%)

*Bolded values represent AEs considered drug-related by the investigator*.

Overall, administration of GSK1440115 at doses up to 750 mg was safe and well-tolerated in asthmatic subjects. A summary of AEs reported in the Phase Ib study is provided in Table 3. In these subjects, there were more AEs reported after administration of GSK1440115 relative to placebo. AEs that were considered drug-related included primarily headache and nausea followed by oral herpes and decreased hemoglobin. A majority of AEs were mild in intensity. There were no deaths or serious AEs during this study. There were no clinically significant findings in clinical laboratory, vital signs or ECGs in this study. There were no dose-related trends in changes in SBP, DBP, MAP, HR, or peak expiratory flow.

**Table 3:** **Summary of adverse events – phase Ib study in asthmatic subjects**.

Adverse event	GSK1440115 (*N* = 12) n (%)	Placebo (*N* = 12) n (%)	All treatments (*N* = 12) n (%)
Subjects with any AE	9 (75.0)	6 (50.0)	10 (83.3)
Nausea	5 (41.7)	1 (8.3)	5 (41.7)
Vomiting	1 (8.3)	0 (0.0)	1 (8.3)
Gastroenteritis viral	1 (8.3)	0 (0.0)	1 (8.3)
Oral herpes	1 (8.3)	0 (0.0)	1 (8.3)
Viral upper respiratory tract infection	0 (0.0)	1 (8.3)	1 (8.3)
Contusion	0 (0.0)	1 (8.3)	1 (8.3)
Laceration	0 (0.0)	1 (8.3)	1 (8.3)
Hemoglobin decreased	1 (8.3)	0 (0.0)	1 (8.3)
Liver function test abnormal	1 (8.3)	0 (0.0)	1 (8.3)
Headache	5 (41.7)	1 (8.3)	6 (50.0)
Presyncope	1 (8.3)	0 (0.0)	1 (8.3)
Rhinorrhea	0 (0.0)	1 (8.3)	1 (8.3)
Wheezing	0 (0.0)	1 (8.3)	1 (8.3)
Dermatitis contact	0 (0.0)	1 (8.3)	1 (8.3)
Eczema	0 (0.0)	1 (8.3)	1 (8.3)

### Pharmacokinetics

In the FTiH study, there was a high degree of variability in the PK data that did not allow for adequate overall assessment of the PK profile for GSK1440155 in healthy volunteers. Exposures of GSK1440115 were more than dose proportional in both the fed and fasted states. A marked food effect was observed in the PK of GSK1440115. At the 750 mg dose, the mean *C*_max_ was 6833 ng/ml, the mean AUC_(0–∞)_ was 35432 ng h/ml and the median *t*_max_ was 8 h. All subjects dosed at the 750 mg dose level achieved concentrations of GSK1440115 that were predicted to be associated with >96% RO based on previous preclinical RO modeling (96% RO was predicted at concentration of 575 ng/ml). All subjects maintained concentration levels above this threshold for at least 3 h (between 4 and 7 h post-dose).

Pharmacokinetics data from the Phase Ib study are summarized in Table 4 which was consistent with the healthy volunteer data at the same 750 mg dose. GSK1440115 was detectable in all asthmatic subjects at 2 h (first post-dose timepoint). *T*_max_ was reached between 2 and 6 h (median: 6 h) and all subjects had measurable concentrations at 24 h post-dose (last PK time point).

**Table 4:** **Summary of pharmacokinetic data after administration of 750 mg dose of GSK1140115 in asthmatic subjects**.

Pharmacokinetics parameters	*n*	Mean (CV)	Median	Geometric mean (CV)	Range (min, max)
*C*_max_ (ng/mL)	12	8473 (55%)	7292	7267 (66%)	(2409, 16605)
t_lag_ (h)	12	0	0	n/e	(0, 0)
*t*_max_ (h)	12	n/e	6.0	n/e	(2,6)
AUC _(0–t)_ (ng h/mL)	12	39340 (65%)	29539	32112 (76%)	(12084, 88351)
AUC _(0-∞)_ (ng h/mL)	12	39365 (65%)	29548	32137 (76%)	(12099, 88421)
λ_z_ (1/h)	12	0.3598 (12%)	0.3564	0.3575 (12%)	(0.287, 0.414)
t_1/2_ (h)	12	1.95 (12%)	1.95	n/e	(1.68, 2.42)

*n/e, not estimated; CV, coefficient of variation; SD, standard deviation*.

### Pharmacodynamics in asthmatics

**Figure 2:**
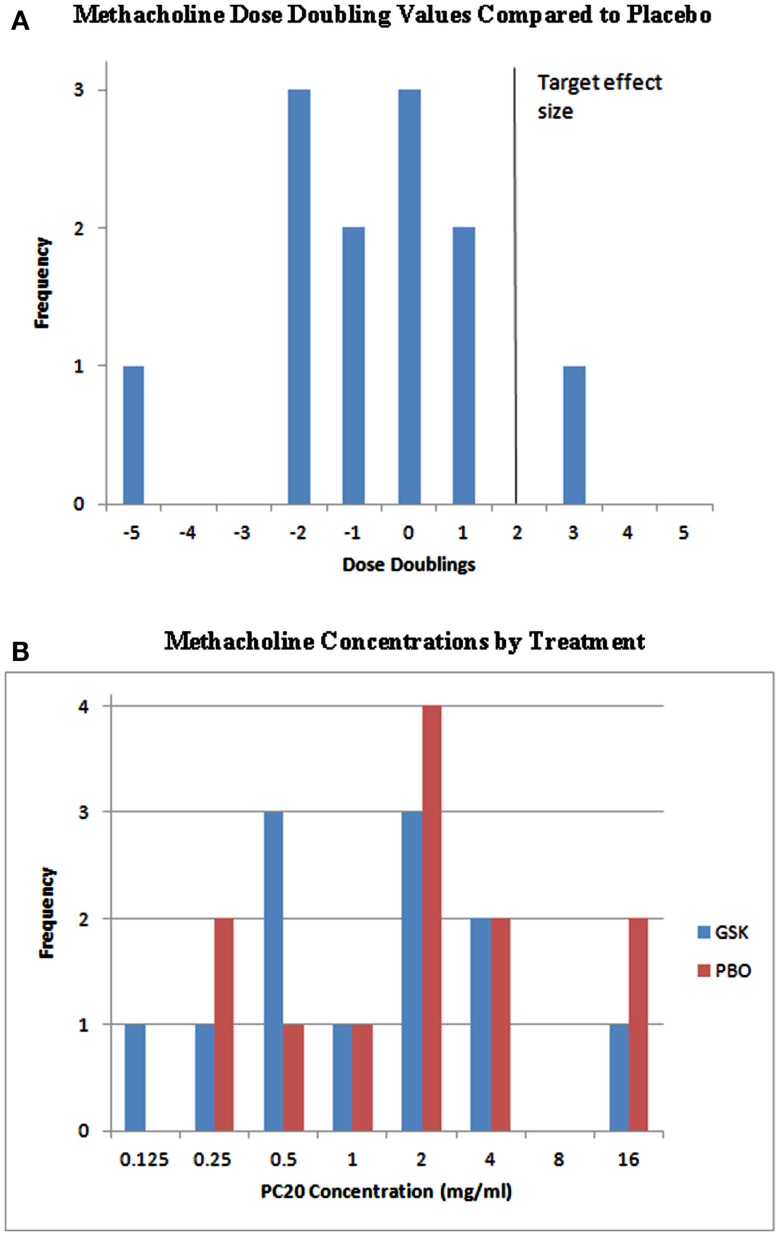
**Individual PC_20_ effects and methacholine dose doublings**. **(A)** The target effect in this study was defined as a mean single double dilution increase (solid vertical line). As shown, the majority of subjects did not have methacholine dose doubling compared to placebo. The probability that the true population mean attained this clinically relevant effect was 0.0237, falling short of ≥0.55 that would be considered effective. **(B)** Metahcholine concentrations presented by treatment. GSK, GSK1440115; PBO, placebo.

For the analysis of methacholine PC_20_, a clinically relevant effect was defined as a mean single double dilution increase beyond placebo; however, this effect was not observed in this study. As shown in Figure 2, the majority of subjects did not have methacholine dose doubling compared to placebo. The probability that the true population mean attained this clinically relevant effect was 0.0237, falling short of ≥0.55 that would be considered effective.

**Figure 3:**
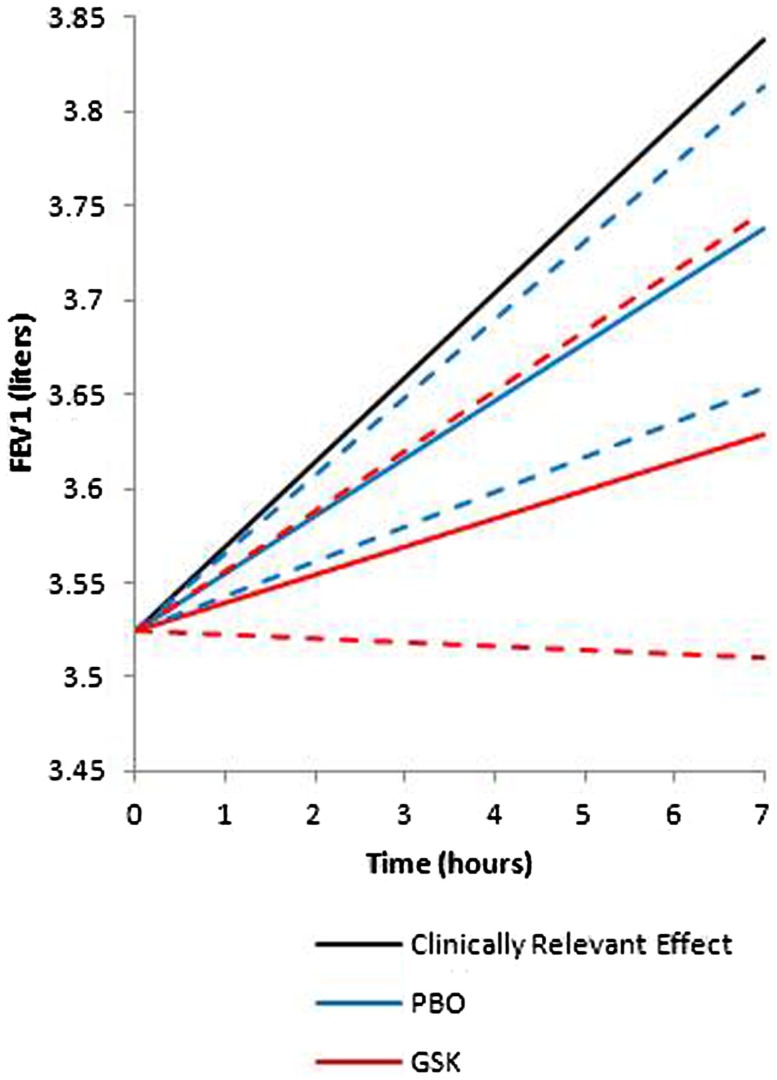
**Mean FEV1 values over time**. A clinically relevant effect was defined as a mean increase in predicted FEV1 of 0.1 l. For both GSK1440115 and placebo the mean FEV1 values (solid lines) over time were below what would be considered a clinically relevant. The probability the true population mean attainted this clinically relevant effect was 0.0014. The 95% credible interval (hashed lines) for the difference in FEV1 beyond placebo was (−0.1276, 0.0508) indicating no significant difference.

The secondary endpoint, FEV1, was analyzed as a continuous variable by treatment for each time point from 0 to 7 h. A clinically relevant effect was defined as a mean increase in predicted FEV1 of 0.1 L. In this study, for both GSK1440115 and placebo, the mean FEV1 values (solid lines) over time were below what would be considered a clinically relevant (Figure 3). The probability the true population mean attainted this clinically relevant effect was 0.0014. The 95% credible interval (hashed lines) for the difference in FEV1 beyond placebo was (−0.1276, 0.0508) indicating no significant difference.

There were no apparent trends or relationships between the systemic plasma exposure of GSK1440115 and either pharmacodynamic endpoint, PC_20_, and FEV1.

## Discussion

Although no information exists on the role of U-II in the control of respiratory physiology in humans, available data in the literature is highly suggestive of a potential role of U-II and its receptor system in bronchial tone in several preclinical species providing a rationale for its evaluation in humans (Hay et al., 2000; De Garavilla et al., 2001; Behm et al., 2005). The purpose of these studies was to investigate the safety, tolerability, and the potential role of a novel UT antagonist, GSK1440115, as a bronchodilator in humans.

Both the Phase I study in healthy volunteers and the Phase Ib proof-of-mechanism study in asthmatics showed that administration of GSK1440115 from 1 to 750 mg was safe and well-tolerated. In both studies, there was high degree of variability in the observed PK following oral dosing with GSK1440115 at all doses. There was a marked food effect in healthy volunteers at the 50 mg dose. In the fasted state, AUC exposures were approximately two-fold lower than when administered with a high fat meal. In the presence of food with the 750 mg dose in the Phase 1b study, the time to maximal concentration was between 2 and 6 h and the terminal half-life was short at approximately 2 h. All subjects maintained greater than the predicted concentration levels necessary to achieve 96% RO for at least 3 h (between 4 and 7 h post-dose). There were no apparent trends or relationships between the systemic plasma exposure of GSK1440115 and either pharmacodynamic endpoint, PC_20_ and FEV1.

Contrary to data observed in preclinical models, the results of the proof-of-mechanism study with GSK1440115 indicated that a U-II receptor antagonist may not be an effective agent as an acute acting oral bronchodilator for treatment of asthma. In asthmatic subjects, 750 mg dose of GSK1440115 lacked any measurable activity as an oral bronchodilator (FEV1) and demonstrated an inability to protect against methacholine-induced bronchoconstriction. Volunteers selected for this trial were required to first demonstrate a minimum baseline PC_20_ in response to methacholine (the concentration of methacholine needed to reduce FEV1 by 20%) and were required to demonstrate a minimum improvement in PC_20_ following an inhaled short acting β-agonist (SABA; ventolin) of at least a two double dilution improvement. Based on the PK profile of the FTiH study in healthy volunteers, in which *T*_max_ was achieved between 4 and 7 h, the methacholine challenge was conducted 7 h after drug administration, a time at which the subjects were expected to have achieved 96% RO. GSK1440115, however, failed to demonstrate any change in PC_20_ in response to a methacholine challenge compared to placebo. The decision rule for this study was based on a posterior probability of a true effect being greater than only 1 double dilution (i.e., a “low hurdle”) and provided a 90% probability that the study would correctly identify an effect if the true effect was in fact two double dilutions (comparable to ventolin). However, based on the PC_20_ data, the probability that GSK1440115 offers an effect size of >1 double dilution beyond placebo in the methacholine challenge model was only 2.37%. Volunteers also demonstrated appropriate changes in FEV1 in response to SABA at screening, but no movement in FEV1 on GSK1440115 was observed during the treatment period compared to placebo. The probability that an effect size of >0.1 L increase in FEV1 over placebo (considered a clinically relevant effect) during the dosing period was 0.14%.

Based upon the available data from these studies it is apparent that antagonism of UT receptor with a single dose of GSK1440115 does not result in bronchodilation. These results suggest that endogenous hU-II does not play a direct role in regulating bronchomotor tone, potentially a result of low UT expression in human bronchiolar tissue (Maguire et al., 2000). Interestingly, it is unknown whether or not UT expression is altered in asthmatic patients. Although the precise reason for the lack of bronchodilator activity in the present study is unidentified, it is unlikely that it is a result of limited drug exposure. Indeed, all subjects dosed with 750 mg GSK1440115 achieved concentrations that were predicted to be associated with >96% RO. This is in contrast to palosuran which exhibits low hUT receptor affinity/activity in intact cells and tissue membranes, offering a potential explanation for the lack of clinical efficacy reported with palosuran in diabetic nephropathy patients (Behm et al., 2008). Preclinical distribution studies with GSK1440115 resulted in wide distribution into the peripheral tissues, including lung, which suggests that access to the UT receptor would not be the limiting factor.

Since the primary goal of this study was to investigate the specific hypothesis of protection against acute bronchospasm following a direct challenge through the use of an oral urotensin antagonist, an alternative challenge modality, such as an allergen associated with late phase inflammatory reactions, was not investigated. Furthermore, a meaningful assessment of allergen associated effects following single dose administration of this agent was not anticipated.

It is important to note that only a single dose of GSK1440115 was tested in this study for a potential acute effect. Although no acute bronchodilator effect was observed in the present study, U-II has been shown to play a predominant role in airway remodeling in asthmatic rats (Liang et al., 2010), suggesting that chronic dosing with GSK1440115 might be useful for preventing or reversing human airway remodeling associated with asthma or other respiratory diseases. Additional studies are required to test this hypothesis.

In summary, GSK1440115 does not induce bronchodilation in asthmatic subjects, suggesting that acute UT antagonism will not be of benefit in this patient population. Although GSK1440115 elicited no bronchodilator effect, acute treatment with the compound was safe and well-tolerated, supporting further use of GSK1440115 as a clinical tool for investigating the role of the U-II system in human disease.

## Conflict of Interest Statement

All authors were employes of GlaxoSmithKline.
